# Editorial: Recent Trends in Preparation, Characterization and Applications of Nanocellulose

**DOI:** 10.3389/fchem.2020.594379

**Published:** 2020-10-19

**Authors:** Djalal Trache, M. Hazwan Hussin, Nicolas Brosse

**Affiliations:** ^1^Teaching and Research Unit of Energetic Processes, Energetic Materials Laboratory, Ecole Militaire Polytechnique, Algiers, Algeria; ^2^Materials Technology Research Group, School of Chemical Sciences, Universiti Sains Malaysia, Minden, Malaysia; ^3^Laboratoire d'Etude et de Recherche sur le MAtériau Bois (LERMAB), Faculté des Sciences et Techniques, Université de Lorraine, Vandœuvre-lès-Nancy, France

**Keywords:** lignocellulosic materials, cellulose nanocrystals, cellulose nanofibrils, nanocomposites, hybrid materials, functionalization

With increasing environmental and ecological concerns due to the use of petroleum-based chemicals and products, the development of novel materials that combine high efficiency, low prices, and minimum environmental impacts is of great value. In this scenario, nanocellulose appears as a remarkable alternative, since it presents outstanding features such as renewability, biocompatibility, high specific surface area, low density, high elastic modulus, dimensional stability, low thermal expansion coefficient, outstanding reinforcing potential, hydrogen-bonding capacity and eco-friendliness (Trache et al., 2020a).

The production of innovative materials for novel and emerging applications from nanocellulose originating from renewable and abundant lignocellulosic materials has earned particular interest. Lignocellulosic materials can be considered as a valuable alternative, a source for biofuels and chemicals, providing an excellent alternative to petroleum (Liao et al., 2020). It has been shown that nanocellulose can be employed to prepare various high-value products with low environmental and societal impact. Nanocellulose has attracted a tremendous level of attention as revealed by the increasing number of scientific contributions and industrial investments in several fields (Trache, 2018; Dufresne, 2019; Naz et al., 2019; Charreau et al., 2020; Lizundia et al., 2020; Trache et al., 2020b; Zhao et al., 2020). The three-dimensional hierarchical structures that constitute these cellulose nanofibers at different scales, the combination of the physicochemical properties of cellulose, together with various advantages of nanomaterials (e.g., a high specific surface area, aspect ratio) open new opportunities in several fields, ranging from electronics to medical applications. For more than two decades, nanocellulose has been one of the most dynamic research fields, with spectacular results and exciting issues to be addressed. Efficient use of nanocellulose offers certain ecological advantages, extraordinary physicochemical features, and high performance. However, the full employment of the intrinsic properties of starting nanoscale materials necessitates continuous development of robust and versatile isolation, synthesis, surface functionalization, and processing procedures to well control assembly over a variety of length scales. It is also crucial to understand the properties of the obtained nanocellulose and its derived materials through efficient characterization methods.

Based on these premises, the present Special Issue in Frontiers in Chemistry, Sections “Green and Sustainable Chemistry” and “Polymer Chemistry,” entitled “Recent trends in preparation, characterization and applications of nanocellulose” aims to further contribute to the momentum of research and development in nanocellulose, by featuring three original research papers as well as three review papers, authored and reviewed by experts in the field.

The first review paper provided a comprehensive overview on the recent researches in the production, modification, and emerging application of nanocellulose, especially cellulose nanocrystals, with a special focus on the reports published within the past 3 years (Trache et al.). A concise background of cellulose, its structural organization, and the nomenclature of cellulosic nanomaterials has been given. The various experimental approaches used to produce nanocellulose as well as their advantages and shortcomings have been discussed. Some emerging applications based on nanocellulose such as nanocomposites, Pickering emulsifiers, wood adhesives, wastewater treatment, and biomedical have been broadly presented. Despite the interesting achievement in the field, it is claimed that further research activities need to be carried out to fill the gaps through the practical transition from laboratory to industrial scales, the decrease of the energy- and time-consumption, as well as the optimization of the production process.

The second review paper discussed the historical development, the fabrication, and functionalization methods of nanocellulose. The current stage and the prospects of the nanocellulose-based flexible energy and hybrid electronic component and devices has been deeply investigated (Dias et al.). It is revealed that functionalized nanocellulose can be used as an alternative to the conventional petroleum-based electronics. However, the main challenge that should be overcome in the future, to fully exploit the potential of nanocellulose and integrate it within materials into society and commerce, is the combination of features such as flexibility, conductivity, luminescence, low environmentally impact, and acceptable cost.

A comprehensive review dealing with nanocellulose-reinforced thermoplastic starch (TPS), polylactic acid (PLA), and polybutylene succinate (PBS) for food packaging applications was also published by Nazrin et al. The authors reported that the loading of nanocellulose into PBS and PLA enhanced their mechanical characteristics and oxygen barrier, whereas nanocomposites containing TPS and nanocellulose presented low water barrier and tensile properties. The authors have also mentioned that the increase in the nanocellulose loading did not compulsory improve their practical features because agglomeration may occur. However, the use of compatibilizers can promote and improve the dispersion through the generation of strong interfacial bonding between nanocellulose and the polymeric matrices, leading to better barrier and mechanical characteristics with biodegradable features, which are widely required for food packaging applications.

The topic of integrated processes in biorefinery, which allows converting biomass into value-added products with maximum energy and material recovery, has attracted considerable interest. To this end, one of the original research papers of this special issue focused on the development of a new biorefinery strategy that introduced the hydrothermal treatment of wood pulp prior to acid hydrolysis (Beyene et al.). The authors demonstrated that 20% of xylan can be transformed into furfural through the hydrothermal treatment, which can also assist the hydrolysis process to generate cellulose nanocrystals with significant yield improvement.

Another interesting research work carried out by Wakabayashi et al. focused on the tailoring of nanocellulose film properties through the control of the degree of fibrillation of 2,2,6,6-tetramethylpiperidine-1-oxyl (TEMPO)-oxidized cellulose (TOC) dispersed in water and disintegrated with a magnetic stirrer or high-pressure homogenizer under various conditions. It is revealed that the fibrillated TOC/water dispersions with a controllable degree of fibrillation allowed obtaining TEMPO-oxidized cellulose nanonetwork films with versatile optical, porous, and mechanical features as well as low oxygen permeability.

Despite the employment of poly(vinyl alcohol) (PVA) hydrogels in several applications and their commercial availability, the investigation of such aerogels supplemented with cellulose nanocrystals (CNC) prepared by the freeze-thrawing method is still within current research focus. Butylina et al. investigated the effect of both PVA and CNC on the characteristics of composite hydrogels prepared by the freeze-traw method. It is proved that the use of 10% of CNC provided a more dense structure with excellent mechanical performance and high-water content, which are considered as crucial features in medical applications such as artificial tissue engineering.

In summary, the present special issue advances not only our understanding of the emerging and important role of nanocellulose in various fields but also challenges and future research directions to fully exploit its fascinating properties in practical ways.

## Author Contributions

All authors listed have made a substantial, direct and intellectual contribution to the work, and approved it for publication.

## Conflict of Interest

The authors declare that the research was conducted in the absence of any commercial or financial relationships that could be construed as a potential conflict of interest.
